# Supplementary material for: Fouling-resistant biofilter of an anaerobic electrochemical membrane reactor
Source: Nat Commun. 2019 Oct 24;10:4860. doi: 10.1038/s41467-019-12838-7 (PMC6813349; doi:10.1038/s41467-019-12838-7)
Supplement: Supplementary file 1 — Supplementary Information [file 41467_2019_12838_MOESM1_ESM.pdf]

Supplementary Information

Title:

Fouling-resistant biofilter of an anaerobic electrochemical membrane reactor

Author: Yu et al

Supplementary Discussion

Conventionally, using membrane as the anode was not considered for the electrostatic adherence in the anode would aggravate membrane fouling. As shown in the results above, although the membrane fouling increased in the initial stage with the electrostatic adherence in the anode membrane, the fouling was finally alleviated by the anode oxidation due to the enrichment of exoelectrogens and the structure changes of the sludge cake layer on the membrane. The sludge cake layer with high electro-activities ensured the anode oxidation of the fouling efficiently to decompose the excessive EPS. With the massive decomposition of EPS of sludge cake layer, the sludge cake layer on the anode membrane became thin and promoted the water flux to lower the TMP, in which the exposed cells eventually formed the mesh-like bio-filter to reserve the interception capacity. In this case, using the membrane as the anode could be a promising strategy to solve the membrane fouling.

Extracellular electron transfer from exoelectrogens to insoluble electrode is a critical process of the anode oxidation of bio-electrochemical systems, which is completed via the following pathways including directly contact, extracellular nanowires such as pili, electron shuttle, et al. The increases of electro-activity of bio-filter in the electron storage capacity and electrical conductivity benefited the extracellular electron transfer to electrode. The polarization of amide groups of the sludge cake layer during the operation of reactor increased the electron storage capacity of the sludge which allowed the amide-based molecules such as proteins to accommodate more electrons participating in anode oxidation, and the polarization of

amide groups also provided more reaction sits in high-energy state as the relay stations for electron transfer. Moreover, the depolarization of the N-H groups accelerated the H-radical coupled electron transfer along the proteins. The changes of the functional EPS could be endogenic such as the secretion by microbes and also be ectogenic such as the polarization by the anode potential. The enhanced electron storage and transfer capacity of the EPS such as proteins could promote the anode oxidation. These functional EPS ensured the efficient decomposition of the membrane fouling, despite massive EPS were degraded.

Supplementary Methods

Membrane Preparation

The procedures for preparing CNT membrane are as below: (1) Surface-functionalized CNTs: 2 g CNTs (DK nano, China), 60 mL H_2SO_4 (98.3%) and 20 mL HNO_3 (78%) were completely mixed in a round-bottomed flask and heated at 80 ℃ under stirring for over 30 min to improve the dispersibility and solubleness in organic solvents. After cooling, the mixture was diluted with 800 mL high pure water and stewing for 12 h. Then the precipitate was filtrated with a 0.45 μm cellulose acetate membrane, and the residue CNTs were dried with a freeze dryer for 24 h. (2) CNTs' coating: 0.05 g acidulated and dried CNTs were scattered in 100 mL organic dimethylformamide solutions with an ultrasonator for 48 h. Then, 30 mL dispersed solution was filtrated with glass microfiber filters (Whatman, England). Finally, the CNT-loaded membranes were dried and calcined at 300 ℃ for 2 h for use. The mean

pore size was 0.55 μm.

Sludge Cake Layer Staining

The fluorescence of SYTO63 (Thermo Fisher, USA) was detected to identify total cells by excitation at 633 nm and emission at 650–700 nm. The fluorescein-isothiocyanate (FITC) (Solarbio, USA) probe was detected to identify proteins by excitation at 488 nm and emission at 500–550 nm. Concanavalin A (con A) (Cayman, USA) conjugates were detected to identify α-polysaccharides by excitation at 543 nm and emission at 550–600 nm. Calcoflour white (CW) (Sigma, Germany) conjugates were detected to identify β-polysaccharides by excitation at 405 nm and emission at 410-480 nm[1,2]. These complexes were imaged using a ×10 objective lens and analyzed using the Olympus confocal software.

The specific staining was conducted on the membranes as described by Chen et al[3,4]. Briefly, SYTO63 (20 μM, 100 μL) was first added to the sample that was placed on a shaker table for 30 min. Next, 0.1 mol L^{-1} $NaHCO_3$ buffer (100 μL) was added to maintain the amine group in non-protonated form. Finally, a solution of FITC (10 g L^{-1}, 10 μL) was added, and the mixture was stirred for 1 h, followed by the addition of con A and calcoflour white (250 mg L^{-1}, 100 μL) for 30 min. After each of the four aforementioned staining stages, the stained sample was washed twice in phosphate buffer saline (PBS) solution to remove the extra probe, and the staining sample was stored at 4 ℃ before any observation.

Microbial Community

After the operation, the memebrane were taken out to analyze the bacterial communities via high-throughput 16S rRNA pyrosequencing. The 16S rRNA gene V4 variable region PCR primers 515/806 were used in a single-step 30 cycle polymerase chain reaction (PCR) with the HotStarTaq Plus Master Mix Kit (Qiagen, USA). The following amplification cycling scheme was used: 94 ℃ for 3 min, followed by 28 cycles of 94 ℃ for 30 s, 53 ℃ for 40 s and 72 ℃ for 1 min, then a elongation step was finally performed at 72 ℃ for 5 min[5,6]. To analyze the microbial community, the sequences obtained were phylogenetically allocated down to the phylum, class and genus level using BLASTN against a curated database derived from GreenGenes, RDPII and NCBI[7].

In situ Fourier Transform Infrared Spectroscopy Spectra

The platinum electrode, the SCE and the platinum sheet were used as the working electrode, the reference electrode and the counter electrode, respectively. A thin layer configuration equipped with a CaF_2 window was placed out of the infrared spectroscopy chamber in a vertical configuration. 0.02 g membrane fouling taken from the sludge cake layers were firstly scattered in 2 mL Nafion solution and then 200 μL mixture were coated on the surface the working electrode. Then the electrodes were placed inside the IR cell that was filled with 0.1 M PBS and 0.1 M KCl to avoid the dosage effect to the spectra. The workstation (CHI 660, Chenhua Instrument, China) was used to control the potential of the working electrode shifting at a range of

−0.8–0.2 V with an interval of 0.2 V[8,9]. Fourier transform infrared spectroscopy (FT-IR) spectra were in the wavenumber range 500-4500 cm^{-1} and the spectra were subsequently analyzed with the OMNIC 8.0 software (Thermo Nicolet). The presence of functional groups was noted by the Spectral Interpretation and literature[10,11].

For two-dimensional correlation spectroscopy (2DCOS), in the synchronous maps, the intensity of the auto peaks represents the sensitiveness of bands to the external perturbation, and the cross-peaks represents whether the orientation of band changes is same. While in the asynchronous maps, the cross-peaks represents the timing sequence of band changes to confirm the homology of the bands.

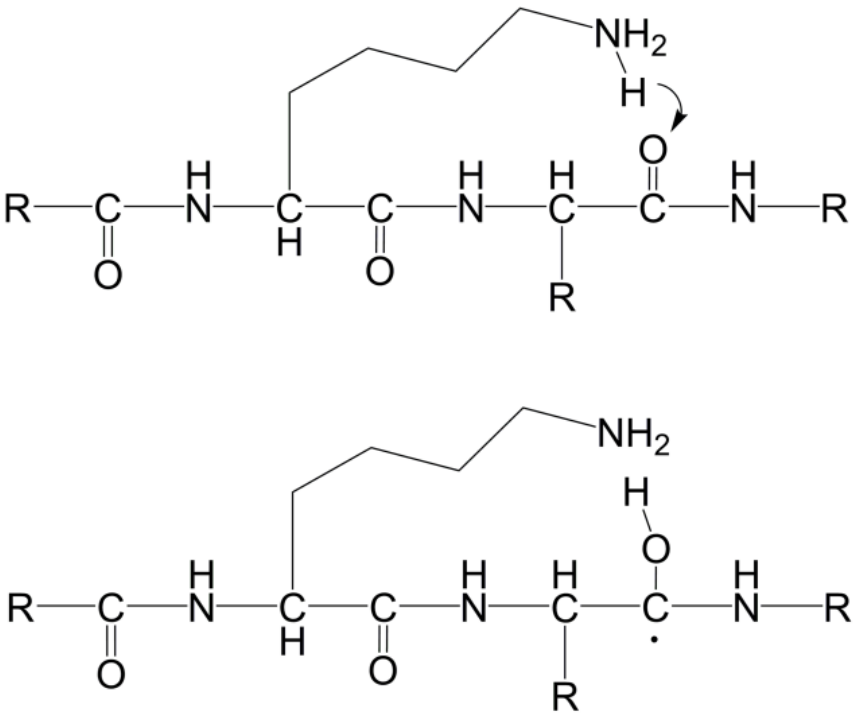

Supplementary Figure 1. A proton-coupled electron transfer model along proteins.

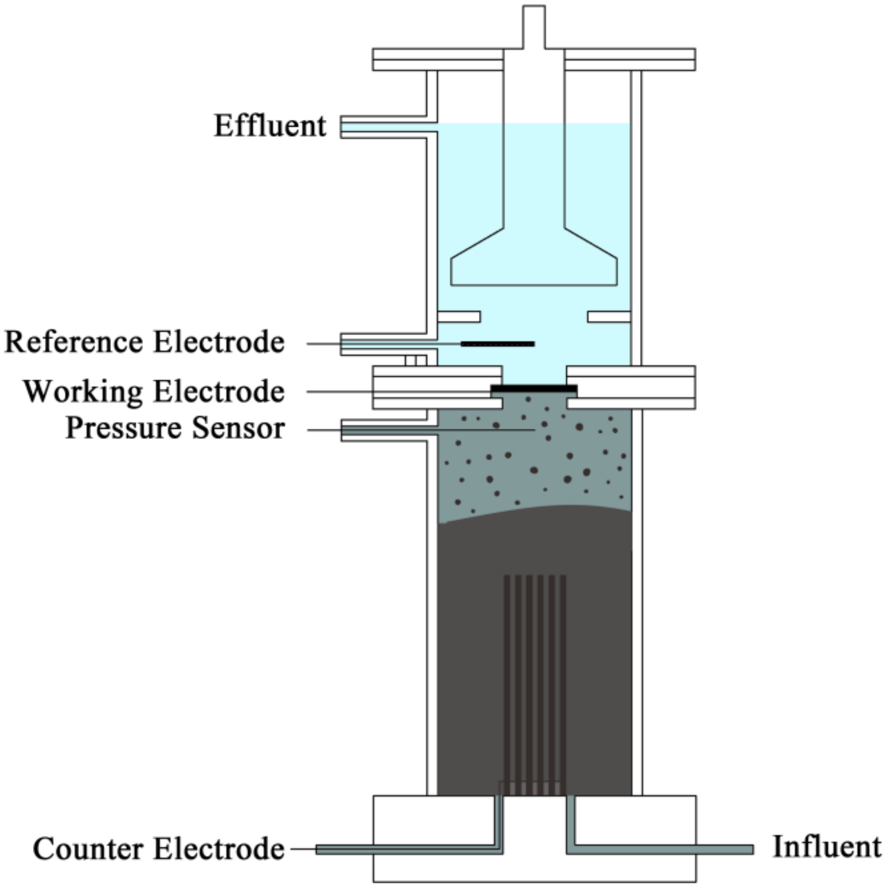

Supplementary Figure 2. Schematic diagram of the reactors.

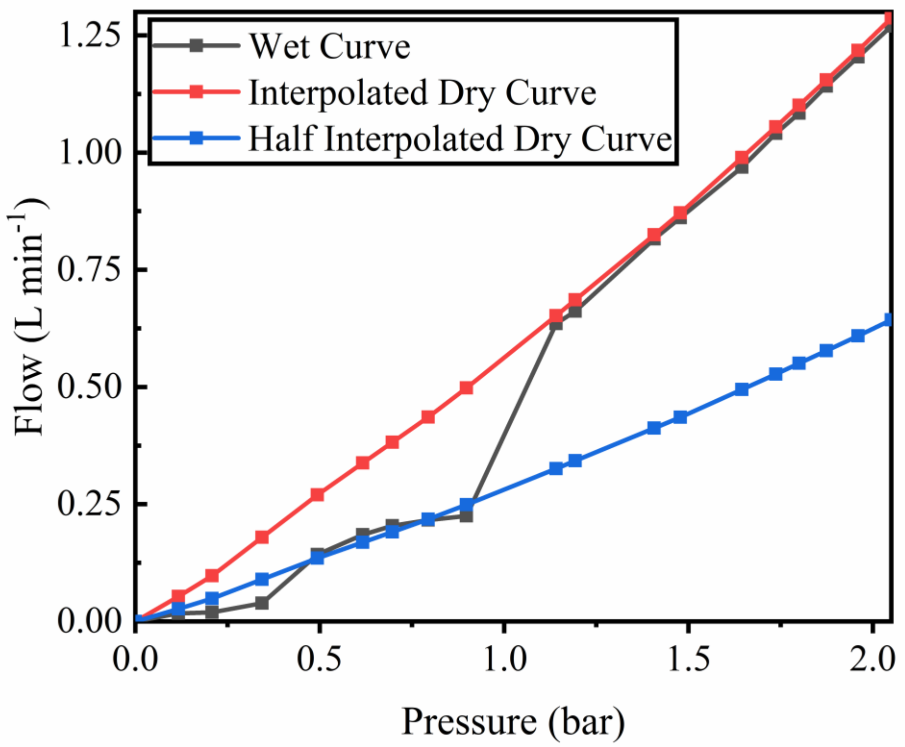

Supplementary Figure 3. Wet and dry curves of the CNT-membrane along with the pressure. (Source data are provided as a Source Data file.)

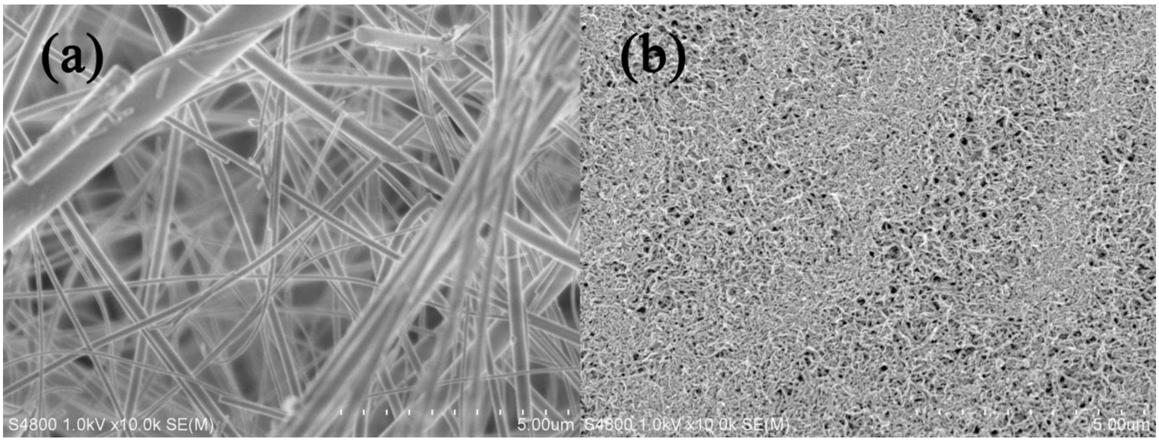

Supplementary Figure 4. Morphologies of (a) the glass microfiber filter and (b) the carbon nano-tube membrane.

Supplementary Table 1. Bacterial community structures of the sludge cake layers of the membranes after operation, The genus level with relative abundance lower than 1.00% were classified into group ‘others’.

Genus	Relative Abundance (%)	
	R-M	R-0.3
Parcubacteria_genera_incertae_sedis	8.29%	12.00%
Longilinea	3.83%	3.88%
Treponema	4.48%	2.41%
Clostridium sensu stricto	1.81%	3.91%
Candidatus Cloacamonas	2.46%	2.72%
Geobacter	4.80%	0.65%
Subdivision3_genera_incertae_sedis	3.27%	1.82%
Bifidobacterium	0.45%	3.31%
Thermogutta	0.87%	2.93%
Smithella	2.27%	1.51%
Ornatilinea	1.55%	1.82%
Methanothrix	0.41%	2.32%
Raoultella	1.78%	1.07%
Levilinea	1.76%	0.86%
Syntrophomonas	0.96%	1.25%
Desulfovibrio	1.07%	0.93%
Anaerolinea	0.89%	0.89%
Candidatus Hydrogenedens	0.66%	0.98%
Phaselicystis	0.53%	1.07%
others	17.05%	14.35%
unclassified	40.81%	39.32%

Supplementary Table 2. 2D FTIR COS results on the assignment and sign of each cross-peak in synchronous and asynchronous maps

Position （cm^{-1}）	Assignment	Synchronous maps*		Asynchronous maps*		Sequences of spectral change
1550	C-N group stretching in amide II	1650<br>+	3300<br>-	1650	3300<br>-	N-H group stretching
1650	C=O group stretching in amide I	1550<br>+	3300<br>-	1550	3300<br>-	↓
3300	N-H group stretching	1550<br>-	1650<br>-	1550<br>+	1650<br>+	N-H and C=O group stretching in amide groups

***** Signs were obtained in the upper-left corner of the maps: +, positive; -, negative.

Supplementary Table 3. Contents of the synthetic wastewater fed to the reactors.

Constituents	Contents
COD (glucose)	2000 mg/L
$NaHCO_3$	1350 mg/L
NH_4Cl	300 mg/L
KH_2PO_4	60 mg/L

Supplementary Table 4. Contents of the complicated wastewater fed to the reactors.

Constituents	Contents
Glucose	1200 mg/L
$NaHCO_3$	1200 mg/L
NH_4Cl	300 mg/L
Sodium oleate	100 mg/L
Peptone	100 mg/L
KH_2PO_4	60 mg/L
Sodium humate	10 mg/L
Sodium polymannuronate	10 mg/L
Yeast extract powder	10 mg/L
Trace mineral solutions	12.5 ml/L
Trace vitamin solutions	5 ml/L

Supplementary Table 5. Contents of the trace mineral solutions.

Constituents	Contents
NTA Trisodium Salt (Free acid)	1.5 g/L
$MgSO_4$	3 g/L
$MnSO_4 \cdot H_2O$	0.5 g/L
NaCl	1.0 g/L
$FeSO_4 \cdot 7\ H_2O$	0.1 g/L
$CaCl_2 \cdot 2\ H_2O$	0.1 g/L
$CoCl_2 \cdot 6\ H_2O$	0.1 g/L
$ZnCl_2$	0.13 g/L
$CuSO_4 \cdot 5\ H_2O$	0.01 g/L
$AlK(SO_4)_2 \cdot 12\ H_2O$	0.01 g/L
H_3BO_3	0.01 g/L
$Na_2MoO_4 \cdot 2\ H_2O$	0.025 g/L
$NiCl_2 \cdot 6\ H_2O$	0.024 g/L
$Na_2WO_4 \cdot 2\ H_2O$	0.025 g/L

Supplementary Table 6. Contents of the trace vitamin solutions.

Constituents	Contents
Biotin	2.0 mg/L
Pantothenic Acid	5.0 mg/L
B-12	0.1 mg/L
p-aminobenzoic acid	5 mg/L
Thioctic Acid (alpha lipoic)	5.0 mg/L
Nicotinic Acid	5.0 mg/L
Thiamin	5.0 mg/L
Riboflavin	5.0 mg/L
Pyridoxine HCl	10 mg/L
Folic Acid	2.0 mg/L

Supplementary References:

[1] Juang, Y., Lee, D. & Lai, J., Fouling layer on hollow-fibre membrane in aerobic granule membrane bioreactor. Journal of the Chinese Institute of Chemical Engineers 39 657 (2008).

[2] Chiu, Z. C., Chen, M. Y., Lee, D. J., Wang, C. H. & Lai, J. Y., Oxygen diffusion in active layer of aerobic granule with step change in surrounding oxygen levels. WATER RES 41 884 (2007).

[3] Chen, M. Y., Lee, D. J. & Tay, J. H., Distribution of extracellular polymeric substances in aerobic granules. APPL MICROBIOL BIOT 73 1463 (2007).

[4] Chen, M., Lee, D., Tay, J. & Show, K., Staining of extracellular polymeric substances and cells in bioaggregates. APPL MICROBIOL BIOT 75 467 (2007).

[5] Caporaso, J. G. *et al.*, Global patterns of 16S rRNA diversity at a depth of millions of sequences per sample. Proceedings of the National Academy of Sciences 108 4516 (2011).

[6] Zhao, Z. *et al.*, Potentially shifting from interspecies hydrogen transfer to direct interspecies electron transfer for syntrophic metabolism to resist acidic impact with conductive carbon cloth. CHEM ENG J 313 10 (2017).

[7] DeSantis, T. Z. *et al.*, Greengenes, a Chimera-Checked 16S rRNA Gene Database and Workbench Compatible with ARB. Appl. Environ. Microbiol. 72 5069 (2006).

[8] You, L. *et al.*, Electrochemical in situ FTIR spectroscopy studies directly extracellular electron transfer of Shewanella oneidensis MR-1. ELECTROCHIM ACTA 170 131 (2015).

[9] Jing, X., Liu, X., Deng, C., Chen, S. & Zhou, S., Chemical signals stimulate Geobacter soli biofilm formation and electroactivity. Biosensors and Bioelectronics 127 1 (2019).

[10] Long, D. A., Infrared and Raman characteristic group frequencies. Tables and charts George Socrates John Wiley and Sons, Ltd, Chichester, Third Edition, 2001. Price £135. J RAMAN SPECTROSC 35 905 (2010).

[11] Rong, X. *et al.*, Interaction of Pseudomonas putida with kaolinite and montmorillonite: A combination study by equilibrium adsorption, ITC, SEM and FTIR. Colloids & Surfaces B Biointerfaces 64 49 (2008).
