# Supplementary material for: Fouling-resistant biofilter of an anaerobic electrochemical membrane reactor
Source: Nat Commun. 2019 Oct 24;10:4860. doi: 10.1038/s41467-019-12838-7 (PMC6813349; doi:10.1038/s41467-019-12838-7)
Supplement: Supplementary file 4 — Reporting Summary [file 41467_2019_12838_MOESM4_ESM.pdf]

nature research

Corresponding author(s): Yaobin Zhang

Last updated by author(s): Sep 28, 2019

# Reporting Summary

Nature Research wishes to improve the reproducibility of the work that we publish. This form provides structure for consistency and transparency in reporting. For further information on Nature Research policies, see Authors & Referees and the Editorial Policy Checklist.

## Statistics

For all statistical analyses, confirm that the following items are present in the figure legend, table legend, main text, or Methods section.

| n/a | Confirmed | |
|---|---|---|
| ☐ | ☒ | The exact sample size (*n*) for each experimental group/condition, given as a discrete number and unit of measurement |
| ☐ | ☒ | A statement on whether measurements were taken from distinct samples or whether the same sample was measured repeatedly |
| ☒ | ☐ | The statistical test(s) used AND whether they are one- or two-sided<br>*Only common tests should be described solely by name; describe more complex techniques in the Methods section.* |
| ☒ | ☐ | A description of all covariates tested |
| ☒ | ☐ | A description of any assumptions or corrections, such as tests of normality and adjustment for multiple comparisons |
| ☒ | ☐ | A full description of the statistical parameters including central tendency (e.g. means) or other basic estimates (e.g. regression coefficient) AND variation (e.g. standard deviation) or associated estimates of uncertainty (e.g. confidence intervals) |
| ☒ | ☐ | For null hypothesis testing, the test statistic (e.g. *F*, *t*, *r*) with confidence intervals, effect sizes, degrees of freedom and *P* value noted<br>*Give P values as exact values whenever suitable.* |
| ☒ | ☐ | For Bayesian analysis, information on the choice of priors and Markov chain Monte Carlo settings |
| ☒ | ☐ | For hierarchical and complex designs, identification of the appropriate level for tests and full reporting of outcomes |
| ☒ | ☐ | Estimates of effect sizes (e.g. Cohen's *d*, Pearson's *r*), indicating how they were calculated |

*Our web collection on statistics for biologists contains articles on many of the points above.*

## Software and code

Policy information about availability of computer code

| | |
|---|---|
| Data collection | In this study, CHI Version 11.04 was used for electrical signal acquisition (current and potential), OPUS version 6.5.97 was used for FTIR spectra acquisition, FV10-ASW Version 03.00.01.15 was used for CLSM Measurement. |
| Data analysis | In this study, ImageJ 1.48 V was used for fluorescence intensity calculation, ZSim Demo 3.30d was used for EIS fitting, 2Dshige version 1.3 was used for FTIR 2DCOS analysis. |

For manuscripts utilizing custom algorithms or software that are central to the research but not yet described in published literature, software must be made available to editors/reviewers. We strongly encourage code deposition in a community repository (e.g. GitHub). See the Nature Research guidelines for submitting code & software for further information.

## Data

Policy information about availability of data

All manuscripts must include a data availability statement. This statement should provide the following information, where applicable:

- Accession codes, unique identifiers, or web links for publicly available datasets
- A list of figures that have associated raw data
- A description of any restrictions on data availability

All data generated or analysed during this study are included in this published article and its supplementary information files. The source data underlying Figs 1a-e, 4a–b, 6a-b, 8a-c, table 1 and table 2 and Supplementary Figure 3 are provided as a Source Data file. The datasets generated during and/or analysed during the current study are available in the Open Science Framework repository, [https://osf.io/u4pwr/].

# Field-specific reporting

Please select the one below that is the best fit for your research. If you are not sure, read the appropriate sections before making your selection.

☐ Life sciences ☐ Behavioural & social sciences ☒ Ecological, evolutionary & environmental sciences

For a reference copy of the document with all sections, see nature.com/documents/nr-reporting-summary-flat.pdf

# Ecological, evolutionary & environmental sciences study design

All studies must disclose on these points even when the disclosure is negative.

| | |
|---|---|
| Study description | Using membrane with -0.30 and -0.20 V (VS Ag/AgCl) anode potential to alleviate membrane fouling in AnMBR system. |
| Research sample | The water samples were taken from the effluent in laboratory-scale AnMBR reactors. And the sludge cake layers formed along operation were acquired after the operation of the AnMBR reactors. |
| Sampling strategy | The water samples were collected every day, and the sludge cake layers were collected after the end of the operation of the AnMBR Reactors. |
| Data collection | Qilin Yu test the water samples and collected datas such as current, potential and COD every day and the data about the sludge cake layers were collected for FTIR, SEM, CV,EIS and CLSM measurement after the operation. |
| Timing and spatial scale | The data were were collected in-situ from the start of the operation to the end every day. |
| Data exclusions | No data were excluded from the analyses. |
| Reproducibility | All attempts to repeat the experiment were successful. |
| Randomization | This is not relevant to this study, for the samples were measured every day with a stable influent. For every day's measurement, the sample size is one. |
| Blinding | Blinding not relevant to this study, for in every day's measurement, the sample size is one. |

Did the study involve field work? ☐ Yes ☒ No

# Reporting for specific materials, systems and methods

We require information from authors about some types of materials, experimental systems and methods used in many studies. Here, indicate whether each material, system or method listed is relevant to your study. If you are not sure if a list item applies to your research, read the appropriate section before selecting a response.

## Materials & experimental systems

| n/a | Involved in the study |
|---|---|
| ☒ | ☐ Antibodies |
| ☒ | ☐ Eukaryotic cell lines |
| ☒ | ☐ Palaeontology |
| ☐ | ☒ Animals and other organisms |
| ☒ | ☐ Human research participants |
| ☒ | ☐ Clinical data |

## Methods

| n/a | Involved in the study |
|---|---|
| ☒ | ☐ ChIP-seq |
| ☒ | ☐ Flow cytometry |
| ☒ | ☐ MRI-based neuroimaging |

## Animals and other organisms

Policy information about studies involving animals; ARRIVE guidelines recommended for reporting animal research

| | |
|---|---|
| Laboratory animals | The study did not involve laboratory animals. |
| Wild animals | The study did not involve wild animals. |
| Field-collected samples | The study did not involve samples collected from the field. |
| Ethics oversight | No ethical approval or guidance was required since the study with microorganisms in the seed sludge was common and safe. |

Note that full information on the approval of the study protocol must also be provided in the manuscript.
